# Association between Add-On Dipeptidyl Peptidase-4 Inhibitor Therapy and Diabetic Retinopathy Progression

**DOI:** 10.3390/jcm10132871

**Published:** 2021-06-28

**Authors:** Eugene Yu-Chuan Kang, Chunya Kang, Wei-Chi Wu, Chi-Chin Sun, Kuan-Jen Chen, Chi-Chun Lai, Tien-Hsing Chen, Yih-Shiou Hwang

**Affiliations:** 1 Department of Ophthalmology, Chang Gung Memorial Hospital, Linkou Medical Center, Taoyuan 333, Taiwan; yckang0321@gmail.com (E.Y.-C.K.); weichi666@gmail.com (W.-C.W.); cgr999@gmail.com (K.-J.C.); chichun.lai@gmail.com (C.-C.L.); 2 College of Medicine, Chang Gung University, Taoyuan 333, Taiwan; arvinsun@cgmh.org.tw; 3 School of Medicine, Medical University of Lublin, 20529 Lublin, Poland; miranda52879@gmail.com; 4 Department of Ophthalmology, Chang Gung Memorial Hospital, Keelung 204, Taiwan; 5 Biostatistical Consultation Center, Chang Gung Memorial Hospital, Keelung 204, Taiwan; 6 Department of Internal Medicine, Division of Cardiology, Chang Gung Memorial Hospital, Keelung 204, Taiwan

**Keywords:** dipeptidyl peptidase-4 inhibitor, diabetes mellitus, diabetic retinopathy, progression

## Abstract

This study aimed to investigate the association of add-on dipeptidyl peptidase-4 inhibitor (DPP4i) therapy and the progression of diabetic retinopathy (DR). In this retrospective population-based cohort study, we examined Taiwanese patients with type 2 diabetes, preexisting DR, and aged ≥40 years from 2009 to 2013. Prescription of DPP4i was defined as a medication possession ratio of ≥80% during the first 6 months. The outcomes included vitreous hemorrhage (VH), tractional retinal detachment, macular edema, and interventions including retinal laser therapy, intravitreal injection (IVI), and vitrectomy. Of 1,767,640 patients, 62,824 were eligible for analysis. After matching, the DPP4i and non-DPP4i groups each contained 20,444 patients. The risks of VH (*p* = 0.013) and macular edema (*p* = 0.035) were higher in the DPP4i group. The DPP4i group also had higher risks of receiving surgical interventions (retinal laser therapy (*p* < 0.001), IVI (*p* = 0.049), vitrectomy (*p* < 0.001), and any surgical intervention (*p* < 0.001)). More patients in the DPP4i group received retinal laser therapy (*p* < 0.001) and IVI (*p* = 0.001) than in the non-DPP4i group. No between-group differences in cardiovascular outcomes were noted. In the real-world database study, add-on DPP4i therapy may be associated with the progression of DR in patients with type 2 diabetes. No additional cardiovascular risks were found. The early progression of DR in rapid glycemic control was inconclusive in our study. The possible effect of add-on DPP4i therapy in the progression of DR in patients with type 2 diabetes requires further research.

## 1. Introduction

Diabetic retinopathy (DR), a common microvascular complication in patients with diabetes, is also a major cause of blindness in working-age adults [1]. The global number of patients with diabetes is estimated to reach 600 million by 2040, one-third of whom are expected to have DR [2]. Severe DR can lead to complications such as vitreous hemorrhage (VH), tractional retinal detachment (RD), and macular edema [3,4]. DR and its complications may require surgical intervention such as retinal laser therapy, intravitreal injection (IVI) of anti-vascular endothelial growth factor, and in some cases vitrectomy [3,4]. This imposes a substantial economic burden on patients with such conditions and their families [5].

Numerous studies have been conducted on preventing or slowing the progression of diabetic complications. A randomized controlled trial reported that appropriate glucose-lowering reduced the risk of cardiovascular diseases, microvascular complications, and all-cause mortality in patients with diabetes [6]. Another randomized controlled trial indicated that intensive glucose control effectively slowed DR progression in patients with type 2 diabetes [7]. Treatment for systemic conditions, such as hypertension and dyslipidemia, has been demonstrated to be associated with a low risk of DR development or progression [7,8].

Dipeptidyl peptidase-4 (DPP4) inhibitors (DPP4i) are a class of oral hypoglycemics, of which the first agent sitagliptin was approved in 2006 by the US Food and Drug Administration [9]. DPP4i suppress the function of DPP4 and indirectly prolong the serum level of glucagon-like peptide-1 (GLP-1), increasing insulin secretion and reducing glucagon secretion from the pancreas [10]. Although a meta-analysis reported that DPP4i exerted a better hypoglycemic effect than α-glucosidase inhibitors [11], other studies have observed associations between its use and an increased risk of heart failure [12,13]. Moreover, another meta-analysis indicated no beneficial association between DPP4i use and all-cause mortality [14]. Regarding DPP4i use in DR, sitagliptin prevented the effect of diabetes on the blood-retinal barrier in male Zucker diabetic fatty rats. Specifically, it improved endothelial function and prevented inflammation, nitrative stress, and apoptosis in animals [15]. However, the association between DPP4i and DR has not been fully characterized [16,17]. The first clinical study of the possible protective effects of DPP4i on DR progression, published in 2016, included 28 patients with type 2 diabetes [18]. A 2018 population-based study by Kim et al. that used data from the South Korean National Health Insurance Service reported a possible association of DPP4i use with an increased risk of DR events early in the treatment phase [19]. Using the same database, Chung et al. found a neutral association between DPP4i use and sulfonylurea added to metformin therapy and the risk of DR progression. The aggravation of DR by DPP4i remains a concern and requires more clinical investigation [20]. In this study, we investigated the association between add-on DPP4i therapy and DR progression in patients with type 2 diabetes and preexisting DR in a real-world setting.

## 2. Materials and Methods

### 2.1. Data Source

This retrospective population-based cohort study was conducted using the Taiwan National Health Insurance (NHI) Research Database (NHIRD) (Center for Biomedical Resources of National Health Research Institutes, Miaoli, Taiwan). More than 99.8% of the population in Taiwan (approximately 23.7 million people as of 2020) is covered by the NHI program, a single-payer system established in March 1995. The NHIRD contains de-identified information including medical claims data. Information on the NHI program and its databases has been described in detail in previous publications [21,22]. The present study was approved by the Chang Gung Memorial Hospital Ethics Institutional Review Board (IRB No. 201800199B1) and adheres to the principles of the Declaration of Helsinki.

### 2.2. Inclusion and Exclusion Criteria

**Figure 1:**
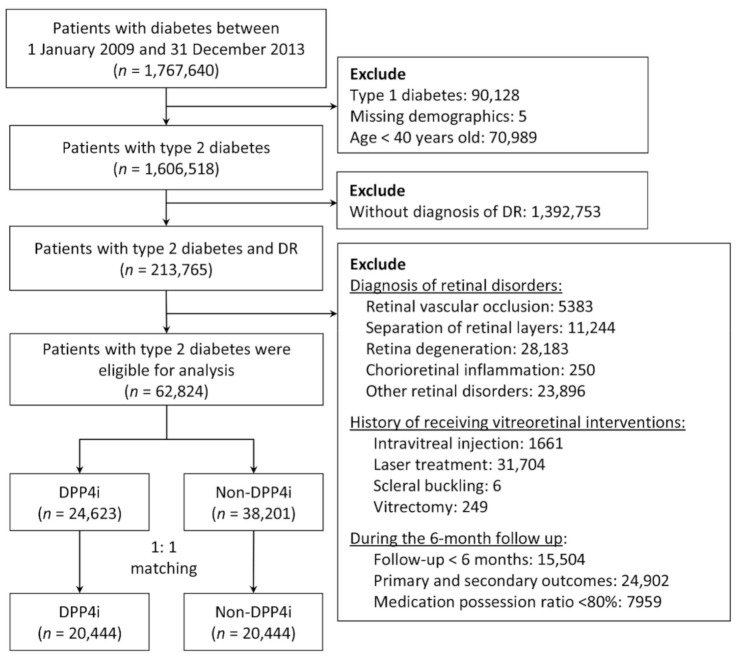
Flowchart of the inclusion and exclusion criteria of the patients. DR, diabetes retinopathy; DPP4i, dipeptidyl peptidase 4 inhibitors.

From 2009 to 2013, we identified patients with diabetes in the NHIRD by using the diagnostic codes of the International Classification of Diseases, Ninth Revision, Clinical Modification (ICD-9-CM). These codes were validated in a study on the accuracy of diabetes diagnosis in NHI claims data. Specifically, at least four outpatient visits for diabetes corresponded to a 95.7% accuracy [23]. Another study observed that a prescription of any oral hypoglycemic agent corresponded to an accuracy of 99% [24]. Therefore, in the present study, we included patients with at least five outpatient diagnoses of type 2 diabetes who were also taking any oral hypoglycemics. Patients with type 2 diabetes and preexisting DR were included in the analysis. We excluded patients who were aged under 40 years as well as those with missing demographic data, type 1 diabetes, retinal disorders (including retinal vascular occlusion, separation of retinal layers, retina degeneration, and chorioretinal inflammation), a history of receiving vitreoretinal interventions (including IVI, retinal laser therapy, scleral buckling, and vitrectomy), or were followed up for less than 6 months (Figure 1).

### 2.3. Group Definition

The index date of the DPP4i group was defined as the date of the first DPP4i prescription between 2009 and 2013. To prevent the immortal time bias, the index date of the non-DPP4i group was assigned as the index date of the DPP4i group through an approach known as prescription time-distribution matching [25]. To ascertain the compliance of DPP4i use, patients in the DPP4i group with a medication possession ratio (MPR) of less than 80% during the first 6 months of follow-up [26], specifically 144 days (180 days × 0.8), were excluded from further analysis (Figure 1).

### 2.4. Outcomes 

In this study, the primary ocular outcome was the composite DR outcome, which consisted of any one of the following: VH, tractional RD, and macular edema. The secondary ocular outcome was the composite outcome of any surgical intervention, namely retinal laser therapy, IVI, and vitrectomy. The cardiovascular outcomes, including myocardial infarction, hospitalization for heart failure, ischemic stroke, and hemorrhagic stroke, were defined as safety outcomes. The primary DR outcome and its components were defined as diagnosis after at least three outpatient diagnoses or one inpatient diagnosis. The surgical interventions and other ocular outcomes were examined using the Taiwan NHI reimbursement codes from the claims data for outpatient and inpatient visits. The occurrence of safety outcomes was determined using the principal discharge diagnosis. Mortality and cardiovascular events selected for analysis have been validated previously [27,28].

### 2.5. Covariates

Covariates were sex, age, proxy variables for compliance (i.e., the number of outpatient visits for diabetes management), proxy variables for DR severity (previous proliferative DR and previous DR duration), comorbidities as well as scores on the Charlson Comorbidity Index, indicators for diabetic severity (diabetes duration, diabetic neuropathy, and diabetic foot ulcer), and concomitant medications. Comorbidities, namely dyslipidemia, hypertension, ischemic heart disease, chronic kidney disease, peripheral arterial disease, ischemic stroke, heart failure, and atrial fibrillation, were confirmed after at least three outpatient diagnoses or one inpatient diagnosis in the previous year. Medications during the first 6 months of follow-up were classified into three categories: antidiabetics, antihypertensives, and other medications. Details of the ICD-9-CM diagnostic codes used in this study are provided in Supplementary Materials (Table S1). The Charlson Comorbidity Index scores were calculated as described previously [29].

### 2.6. Statistics

To reduce confounding effects, the analysis of differences in outcomes between the DPP4i and non-DPP4i groups was performed after propensity score matching (PSM). The propensity score was the predicted probability given the value of the covariates, which was calculated using a multivariable logistic regression model in which the study groups (1: DPP4i and 0: non-DPP4i) were regressed on the selected covariates. The matching was processed using a greedy nearest-neighbor algorithm with a caliper of 0.2 times the standard deviation of the logit of the propensity score. The matching order was random, and replacement was not allowed. Each patient in the DPP4i group was matched with a non-DPP4i control. The matching quality was assessed after PSM by using the absolute value of the standardized difference between the groups, where a value of less than 0.1 was considered negligible.

The Fine–Gray subdistribution hazard model, which considers all-cause mortality a competing risk, was used to compare the occurrence of time-to-event outcomes between the groups. The average number of surgical interventions per decade was also analyzed and compared using the Poisson model, in which the natural logarithm of the follow-up duration was an offset variable. The study groups (DPP4i vs. non-DPP4i) were the only explanatory variable in the regression analysis. The within-pair clustering of outcomes after PSM was accounted for by using robust standard errors through the generalized estimating equation approach [30]. Further subgroup analyses were conducted to evaluate the consistency of the observed treatment effect on the specified outcomes across different levels of subgroup variables. The outcomes of interest comprised the primary and secondary endpoints, namely the composite DR outcome and the composite outcome of any surgical intervention, respectively. The selected subgroups were sex, age (dichotomized at 65 years), previous proliferative DR, hypertension, dyslipidemia, ischemic heart disease, ischemic stroke, chronic kidney disease, peripheral arterial disease, diabetes duration (dichotomized at 10 years), diabetic neuropathy, diabetic foot ulcer, and the use of concomitant antidiabetics (e.g., metformin, sulfonylurea, thiazolidinediones, alpha-glucosidase inhibitors, meglitinides, and insulin). A two-sided *p*-value of <0.05 was considered to be significant. All analyses were performed using SAS software, Version 9.4 of the SAS System (SAS Institute Inc., Cary, NC, USA), including the % cif macro for generating cumulative incidence functions under the Fine–Gray sub-distribution hazard method.

## 3. Results

### 3.1. Participants

Between 2009 and 2013, a total of 1,767,640 patients with diabetes were identified. After the exclusion of patients aged under 40 years as well as those with type 1 diabetes, missing demographic data, and no DR diagnosis, 213,765 patients remained. We further excluded patients who were followed up for less than 6 months or developed any of the primary or secondary ocular outcomes within 6 months after the index date, as well as those with retinal disorders, a history of receiving vitreoretinal interventions or who had an MPR of less than 80%. After these procedures, 62,824 patients remained. After 1:1 PSM, the non-DPP4i and DPP4i groups comprised 20,444 patients each (Figure 1).

### 3.2. Demographic Characteristics

Table 1 presents the demographic characteristics of the study groups before and after matching. Before matching, the patients in the DPP4i group were younger; had more outpatient visits for diabetes management in the previous year; were more likely to have undergone a dilated fundus examination in the previous year; had a higher prevalence of dyslipidemia; had a longer diabetes duration; had more prescriptions of sulfonylurea, alpha-glucosidase inhibitors, meglitinides, beta-blockers, angiotensin-converting enzyme inhibitors/angiotensin II receptor blockers, antiplatelets, statins, and fenofibrates, and fewer prescriptions of insulin. After matching, the two groups were well balanced in terms of sex, age, comorbidities, indicators for diabetic severity, underlying ocular diseases, medications, and follow-up duration.

### 3.3. Primary Ocular Outcomes

**Figure 2:**
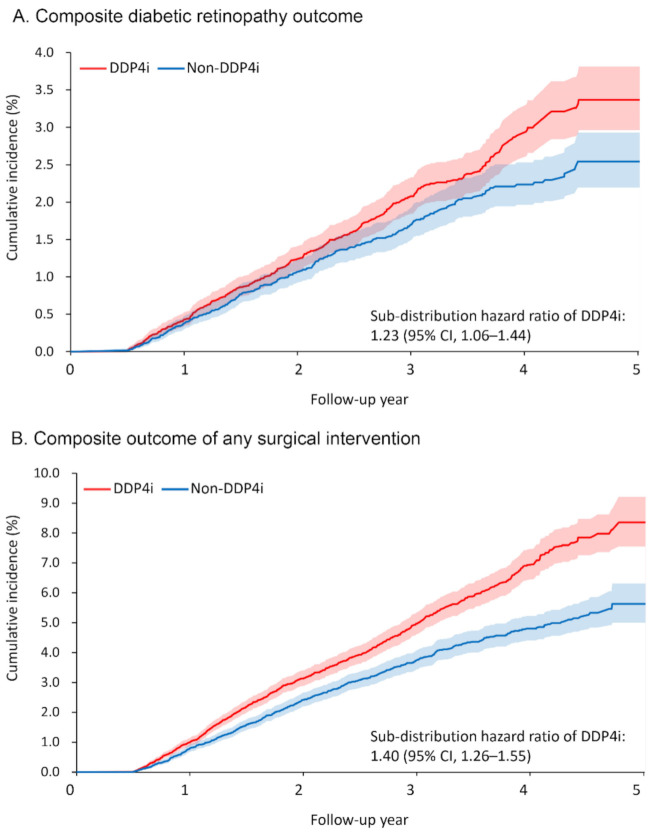
Cumulative incidence function of (**A**) composite diabetic retinopathy outcome and (**B**) composite outcome of any surgical intervention between the DPP4i and non-DPP4i group after propensity score matching. DPP4i, dipeptidyl peptidase 4 inhibitor; CI, confidence interval.

Table 2 presents the primary ocular outcomes of the patients, including any surgical intervention taken. Over a mean follow-up duration of 2.5 years, 366 and 294 patients (1.8% and 1.4%, respectively) in the DPP4i and non-DPP4i groups developed the primary ocular outcome, namely the composite DR outcome. The risk of developing the composite DR outcome was significantly higher in the DPP4i group (sub-distribution hazard ratio [SHR] 1.23, 95% confidence interval [CI] 1.06–1.44; Figure 2A). Among the individual components of the composite DR outcome, the risks of VH (SHR 1.24, 95% CI 1.05–1.48) and macular edema (SHR 1.48, 95% CI 1.03–2.13) were significantly higher in the DPP4i group. 

The DPP4i group also had a higher risk of receiving surgical intervention for severe DR or its complications (retinal laser therapy: SHR 1.75, 95% CI 1.33–2.30; IVI: SHR 1.32, 95% CI 1.001–1.74; vitrectomy: SHR 1.32, 95% CI 1.24–1.40; any surgical intervention: SHR 1.40, 95% CI 1.26–1.55; Figure 2B). As for the number of interventions, more patients in the DPP4i group received retinal laser therapy (rate ratio (RR) 1.39, 95% CI 1.23–1.58) and IVI (RR 1.84, 95% CI 1.28–2.63) than in the non-DPP4i group.

### 3.4. Safety Outcomes

The results of the safety outcomes are shown in Table 3. No between-group differences were observed in any of the safety outcomes, namely myocardial infarction, hospitalization for heart failure, ischemic stroke, hemorrhagic stroke, and the composite outcome of major adverse cardiovascular events.

### 3.5. Subgroup Analysis

**Figure 3:**
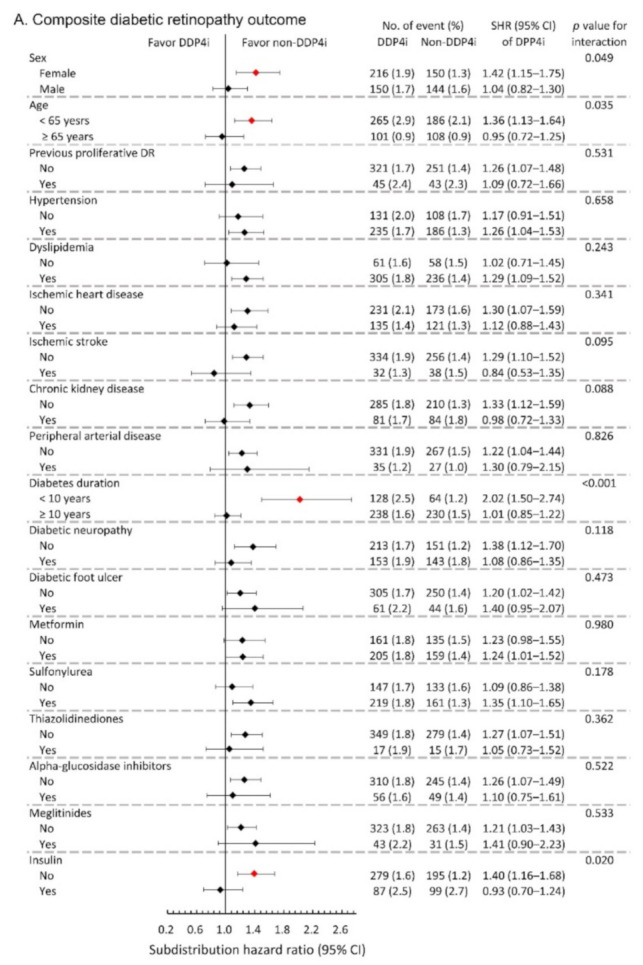

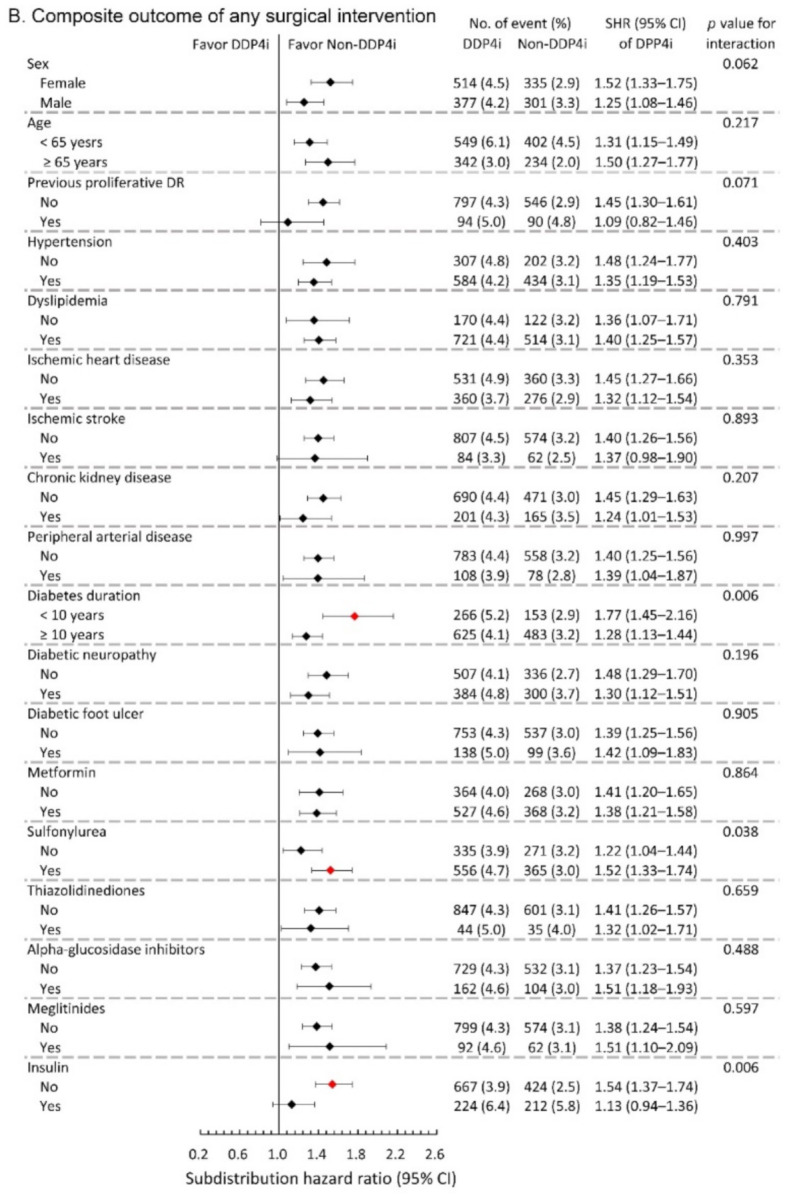
Subgroup analysis of (**A**) composite diabetic retinopathy outcomes and (**B**) composite outcome of any surgical interventions of the diabetic patients with DR between the DPP4i users and non-DPP4i controls in the propensity score-matched cohort. The red color indicates a statistical significance.

We further conducted subgroup analysis on the primary composite DR outcome and the composite outcome of any surgical interventions. The results showed that the observed hazardous effect of DPP4i on the risk of primary composite DR outcome was particularly obvious in the following population: females, younger patients, patients with relatively shorter diabetes duration, and those without taking insulin (All *p*-values for interaction <0.05; Figure 3A). Similarly, the observed increased risk of the composite outcome of any surgical interventions due to DPP4i was more apparent in patients with relatively shorter diabetes duration, and those who took sulfonylurea, and those without insulin therapy (All *p*-values for interaction <0.05; Figure 3B).

## 4. Discussion

The use of DPP4i in glucose-lowering for diabetes has increased considerably over the past decade after being introduced in 2006 [31]. To reduce mortality and morbidity, measuring drugs’ protective effects and related diabetes complications are essential. As mentioned, DR, a major microvascular complication in diabetes, can cause severe visual impairment. Thus, in this population-based study, we evaluated the association between the add-on DPP4i therapy and the progression of preexisting DR in patients with type 2 diabetes aged ≥ 40 years. During the 2.5-year follow-up, the add-on DPP4i therapy was associated with increased risks of composite DR outcome and needs of surgical interventions. However, it did not increase the risk of cardiovascular events.

The association between DPP4i and DR remains a matter of contention in the literature. A study including 82 patients with type 2 diabetes reported that DPP4i use had protective effects on DR progression [18]. A study using a cohort representative of individuals in the US population aged ≥ 65 years observed that DPP4i use had a neutral effect on DR [32]. Some other studies have found that DPP4i cause adverse retinal outcomes. In the Trial Evaluating Cardiovascular Outcomes With Sitagliptin (TECOS), DR occurred more frequently in patients under add-on sitagliptin therapy than in those who were not (2.8% vs. 2.2%) [33]. Another study, using a sample representative of the South Korean population, also indicated an increased risk of DR in early DPP4i treatment (<12 months) [19]. These findings indicate that the pharmacodynamic or effects of DPP4i may vary with population or patient characteristics. 

The non-DPP4i and DPP4i groups in the present study comprised 20,444 patients (after matching) with type 2 diabetes (mean duration of 11 years since onset) and preexisting DR, respectively. VH and macular edema occurred significantly more frequently in the DPP4i group than in the non-DPP4i group. Furthermore, patients in the DPP4i group under add-on DPP4i therapy for diabetes control were more likely to receive surgical intervention for advanced DR. In short, add-on DPP4i therapy increased the risk of DR progression. However, no significant between-group differences in safety outcomes were noted. In addition, DPP4i was not associated with an increased risk of cardiovascular events.

Although the exact mechanism remains uncertain, biochemical changes in retinal cells after DPP4i administration in experimental studies have been inconsistent. Numerous laboratory studies have reported the protective effects of DPP4i on retinal health. For example, Gonçalves et al. found that sitagliptin had an antioxidative effect on rat retinas [34]. In another of their studies, sitagliptin ameliorated bovine retinal endothelial dysfunction caused by inflammation [15]. Another study noted that linagliptin had anti-angiogenic effects on mice with oxygen-induced retinopathy [35]. However, Lee et al. indicated that DPP4i caused disruptions in endothelial cell-to-cell junctions by accumulating stromal cell-derived factor 1α and phosphorylating vascular endothelial cadherin, as well as further increasing retinal vascular permeability [36]. In a 2020 experimental study, the results revealed that prolonged DPP4 inhibition destabilized the blood-retina barrier, potentially inducing retinal edema [37]. Early deterioration of DR was also reported in a GLP-1 analog, semaglutide, although the pharmacodynamic may be different with the DPP4i [38]. Retinal changes under DPP4i therapy may depend on the duration of DPP4i treatment and the severity of diabetes and its complications. Long-term administration of DPP4i in patients with preexisting DR might induce the development of excess vasculature as well as vascular permeability, potentially contributing to exudate production and further exacerbating DR. Thus, more awareness of DR progression may be necessary for patients under long-term DPP4i treatment. 

Cardiovascular complications of DPP4i remain the topic of an ongoing debate. Some studies have reported a decreased risk of cardiovascular events after DPP4i therapy [39,40]. By contrast, other studies have indicated that DPP4i use increased the risk of cardiovascular disorders [17,41]. In our study, the safety outcomes (including myocardial infarction, heart failure, ischemic stroke, hemorrhagic stroke, and composite cardiovascular outcomes) did not differ significantly between the groups. This is consistent with the assessment from the TECOS [33], the Examination of Cardiovascular Outcomes with Alogliptin versus Standard of Care, and the Saxagliptin Assessment of Vascular Outcomes Recorded in Patients with Diabetes Mellitus (SAVOR)—Thrombolysis in Myocardial Infarction (TIMI) [12]. Thus, our additional finding also supported a neutral association between DPP4i use and the occurrence of major adverse cardiovascular events.

A limited number of large-scale clinical studies have evaluated the association of DPP4i and the progression of retinopathy in patients with diabetes. The strength of the present study is that, to the best of our knowledge, it is the first observational investigation of the association of an add-on DPP4i in DR progression in a population-based cohort. By systemically assessing the possible confounding factors and making adjustments through PSM, we minimized detection bias and balanced the clinical characteristics between the groups. The approximately 2.5-year follow-up also means that the present findings demonstrate the long-term impacts—as opposed to the short-term effects—of DPP4i use. The potential harm that may accompany DPP4i use indicated in the present study raises substantial concerns regarding its safe use as an antidiabetic.

This study has some limitations. First, because only patients older than 40 years were included, the present findings cannot be extrapolated to other age groups. Moreover, because the patients were all Taiwanese, it remains unclear whether our findings are generalizable to other populations. Second, we could not completely prevent confounding effects. Nevertheless, we performed matching by systematically considering various variables, minimizing any imbalance between the groups. Third, we could not obtain information on the patients’ diabetes control, as well as the hypoglycemic events, which are important factors of diabetes management. Nevertheless, we have matched the patients in the two groups based on their hypoglycemic agent use. Fourth, data on laboratory tests, such as the serum glucose level or hemoglobin A1c, are not available in the NHIRD. Rapid reduction of hemoglobin A1c may affect early worsening of DR [8]. However, this phenomenon should be counterbalanced in a long-term observation in the patients with better glucose control, which has been reported in the Semaglutide Unabated Sustainability in Treatment of Type 2 Diabetes (SUSTAIN) study [38]. In our study, the follow-up period of 2.5 years is comparable with the previous study, and our case number (*n* = 20,444 in both study and control groups) is higher than the SUSTAIN study (*n* = 8105 across the SUSTAIN 1 to 6 studies) [38]. Whether the DR progression in the add-on DPP4i use is related to rapid hypoglycemic response needs further study. Fifth, the between-patient variation in diabetes severity (with some patients in severe condition) means that the alleviation of systemic disorders with medications remains challenging. The blood pressure change in our study was also not available. Nevertheless, we have matched the groups according to their disease duration, complications, medications, and underlying conditions. Therefore, the clinical characteristics of patients in the two groups were comparable at least in theory. Last, the database did not contain the results of ocular exams including optical coherence tomography, which is essential to differentiate the involvement of diabetic macular edema. The association of DPP4i and the involvement of diabetic macular edema may need further investigations. A prospective randomized trial may be required for understanding the possible effect of add-on DPP4i therapy in the progression of DR in patients with type 2 diabetes.

## 5. Conclusions

In conclusion, add-on DPP4i therapy may be associated with the progression of preexisting DR in patients with type 2 diabetes aged ≥40 years, but the cause and effect need further research DPP4i therapy did not increase the risk of cardiovascular events. Therefore, when choosing hypoglycemic treatments for patients with diabetes and preexisting DR, the possible promoting effect of DPP4i on DR progression should be considered. A close retinal evaluation may be necessary for long-term DPP4i administration.

## Figures and Tables

**Table 1:** Characteristics of patients with type 2 diabetes and diabetic retinopathy before and after matching. Balance achieved between the DPP4i and non-DPP4i groups after matching.

	before Matching			after Matching		
Variable	DDP4i (*n* = 24,623)	Non-DDP4i (*n* = 38,201)	STD	DDP4i (*n* = 20,444)	Non-DDP4i (*n* = 20,444)	STD
Sex (male)	10,745 (43.6)	17,084 (44.7)	−0.02	8936 (43.7)	9013 (44.1)	−0.01
Age (years)	66.5 ± 10.5	68.0 ± 11.0	−0.14	66.7 ± 10.5	66.7 ± 10.8	<0.01
Age ≥ 65 years	13,606 (55.3)	22,849 (59.8)	−0.09	11,416 (55.8)	11,448 (56.0)	<0.01
No. of outpatient visit in the prior year	16.8 ± 8.9	14.1 ± 9.0	0.31	16.1 ± 8.3	16.1 ± 9.7	<0.01
Previous proliferative DR	2195 (8.9)	3605 (9.4)	−0.02	1862 (9.1)	1879 (9.2)	<0.01
Duration of DR (years)	6.1 ± 3.5	6.0 ± 3.5	0.03	6.0 ± 3.4	6.0 ± 3.5	<0.01
Comorbidity						
Dyslipidemia	20,277 (82.3)	29,405 (77.0)	0.13	16,560 (81.0)	16,673 (81.6)	−0.01
Hypertension	17,202 (69.9)	25,236 (66.1)	0.08	14,038 (68.7)	14,140 (69.2)	−0.01
Ischemic heart disease	11,746 (47.7)	17,433 (45.6)	0.04	9626 (47.1)	9608 (47.0)	<0.01
Chronic kidney disease	6126 (24.9)	8035 (21.0)	0.09	4724 (23.1)	4745 (23.2)	<0.01
Peripheral arterial disease	3350 (13.6)	5480 (14.3)	−0.02	2804 (13.7)	2739 (13.4)	0.01
Ischemic stroke	3015 (12.2)	4989 (13.1)	−0.02	2526 (12.4)	2512 (12.3)	<0.01
Heart failure	1470 (6.0)	2464 (6.5)	−0.02	1186 (5.8)	1171 (5.7)	<0.01
Atrial fibrillation	882 (3.6)	1459 (3.8)	−0.01	716 (3.5)	699 (3.4)	<0.01
Charlson Comorbidity Index score	2.5 ± 1.7	2.3 ± 1.8	0.07	2.4 ± 1.7	2.4 ± 1.8	<0.01
Indicator for diabetic severity						
Diabetes duration, years	11.3 ± 2.7	11.0 ± 3.0	0.11	11.2 ± 2.8	11.2 ± 2.9	−0.01
Diabetic neuropathy	9887 (40.2)	14,112 (36.9)	0.07	7980 (39.0)	8065 (39.4)	−0.01
Diabetic foot ulcer	3366 (13.7)	5152 (13.5)	0.01	2762 (13.5)	2751 (13.5)	<0.01
Antidiabetics						
Sulfonylurea	14,543 (59.1)	19,954 (52.2)	0.14	11,921 (58.3)	12,065 (59.0)	−0.01
Metformin	13,162 (53.5)	22,197 (58.1)	−0.09	11,396 (55.7)	11,537 (56.4)	−0.01
Alpha-glucosidase inhibitors	4636 (18.8)	4,779 (12.5)	0.17	3514 (17.2)	3490 (17.1)	<0.01
Thiazolidinediones	3076 (12.5)	210 (13.6)	−0.03	2683 (13.1)	2812 (13.8)	−0.02
Meglitinides	2574 (10.5)	2918 (7.6)	0.10	1996 (9.8)	2024 (9.9)	<0.01
Insulin	3873 (15.7)	8299 (21.7)	−0.15	3488 (17.1)	3633 (17.8)	−0.02
Antihypertensives						
Angiotensin-converting enzyme inhibitors/angiotensin II receptor blockers	15,630 (63.5)	20,002 (52.4)	0.23	12,445 (60.9)	12,577 (61.5)	−0.01
Calcium channel blockers	8509 (34.6)	14,036 (36.7)	−0.05	7174 (35.1)	7213 (35.3)	<0.01
Beta blockers	7654 (31.1)	9780 (25.6)	0.12	6048 (29.6)	6023 (29.5)	<0.01
Alpha blockers	1403 (5.7)	2154 (5.6)	<0.01	1163 (5.7)	1176 (5.8)	<0.01
Thiazide	1075 (4.4)	1545 (4.0)	0.02	886 (4.3)	866 (4.2)	<0.01
Other medications						
Antiplatelets	8767 (35.6)	11,115 (29.1)	0.14	6970 (34.1)	7074 (34.6)	−0.01
Anticoagulants	380 (1.5)	473 (1.2)	0.03	304 (1.5)	284 (1.4)	0.01
Statins	10,788 (43.8)	12,319 (32.2)	0.24	8381 (41.0)	8346 (40.8)	<0.01
Fenofibrates	2552 (10.4)	2894 (7.6)	0.10	1975 (9.7)	1972 (9.6)	<0.01
Follow-up (years)	2.5 ± 1.3	2.4 ± 1.1	0.06	2.6 ± 1.2	2.5 ± 1.2	0.08

DDP4i, dipeptidyl peptidase 4 inhibitor; STD, standardized difference; DR, diabetic retinopathy. Data are presented as frequency (percentage) or mean ± standard deviation.

**Table 2:** Primary ocular outcomes, including any surgical intervention taken, of patients with type 2 diabetes and diabetic retinopathy demonstrating significantly higher risks of composite diabetic retinopathy and surgical interventions in the DPP4i group.

	DDP4i	Non-DDP4i	DPP4i vs. Non-DPP4i	
Outcome	(*n* = 20,444)	(*n* = 20,444)	SHR (95% CI)	*p*-Value
Primary ocular outcome (composite DR outcome)	366 (1.8)	294 (1.4)	1.23 (1.06–1.44)	0.008
Individual component of composite DR outcome				
VH	292 (1.4)	232 (1.1)	1.24 (1.05–1.48)	0.013
Tractional RD	50 (0.24)	35 (0.17)	1.41 (0.91–2.17)	0.122
Macular edema	72 (0.35)	48 (0.23)	1.48 (1.03–2.13)	0.035
Surgical intervention				
Retinal laser therapy	824 (4.0)	582 (2.8)	1.75 (1.33–2.30)	<0.001
IVI	140 (0.68)	79 (0.39)	1.32 (1.001–1.74)	0.049
Vitrectomy	118 (0.58)	88 (0.43)	1.32 (1.24–1.40)	<0.001
Composite outcome of any surgical intervention	891 (4.4)	636 (3.1)	1.40 (1.26–1.55)	<0.001
Number of interventions per 10 years			RR (95% CI) *	*p*-value
Retinal laser therapy	0.6 ± 3.4	0.4 ± 2.9	1.39 (1.23–1.58)	<0.001
IVI	0.06 ± 0.94	0.03 ± 0.67	1.84 (1.28–2.63)	0.001
Vitrectomy	0.03 ± 0.42	0.02 ± 0.38	1.29 (0.94–1.79)	0.117

DDP4i, dipeptidyl peptidase 4 inhibitor; SHR, sub-distribution hazard ratio; CI, confidence interval; RD, retinal detachment; DR, diabetic retinopathy; RR, rate ratio; VH, vitreous hemorrhage; IVI, intravitreal injection. * Estimated using a Poisson model in which the logarithm of follow-up duration was treated as an offset variable.

**Table 3:** Safety outcomes of patients with type 2 diabetes and diabetic retinopathy showing no significant risk in both groups.

	DDP4i	Non-DDP4i	DPP4i vs. Non-DPP4i	
Outcome	(*n* = 20,444)	(*n* = 20,444)	SHR (95% CI)	*p*-Value
Myocardial infarction	252 (1.2)	268 (1.3)	0.93 (0.78–1.10)	0.396
Hospitalization for heart failure	495 (2.4)	441 (2.2)	1.11 (0.98–1.26)	0.115
Ischemic stroke	872 (4.3)	839 (4.1)	1.02 (0.93–1.13)	0.621
Hemorrhagic stroke	131 (0.64)	151 (0.74)	0.85 (0.68–1.08)	0.183
Major adverse cardiovascular events *	1600 (7.8)	1529 (7.5)	1.05 (0.98–1.12)	0.198

DDP4i, dipeptidyl peptidase 4 inhibitor; SHR, sub-distribution hazard ratio; CI, confidence interval. * Any one of myocardial infarction, heart failure, or stroke.

## Data Availability

The data used for the current study cannot be made publicly available according to the NHIRD regulations of personal data protection, allowing only the person responsible for the data management to approach the data after approval from Taiwan NHI bureau.

## Supplementary Materials

The following are available online at https://www.mdpi.com/article/10.3390/jcm10132871/s1, Table S1: ICD-9 CM diagnostic codes used in the study.
