# Supplementary material for: Association between Add-On Dipeptidyl Peptidase-4 Inhibitor Therapy and Diabetic Retinopathy Progression
Source: J Clin Med. 2021 Jun 28;10(13):2871. doi: 10.3390/jcm10132871 (PMC8269314; doi:10.3390/jcm10132871)
Supplement: Supplementary file 1 [file jcm-10-02871-s001.zip › jcm-1267030-supplementary.pdf]

Table S1. ICD-9 CM diagnostic codes

Variable	ICD-9 CM Code
Diabetes mellitus	250.xx
Type 1 diabetes mellitus	25001, 25003, 25011, 25013, 25021, 25023, 25031, 25033, 25041, 25043, 25051, 25053, 25061, 25063, 25071, 25073, 25081, 25083, 25091, 25093
Diabetic retinopathy	362.0x
Retinal vascular occlusion	362.3x
Separation of retinal layers	361.xx, 362.4x
Retina degeneration	362.5x, 362.6x, 362.7x
Chorioretinal inflammation	363.0x, 363.1x, 363.2x
Other retinal disorders	362.8x, 379.23
Proliferative diabetic retinopathy	362.02
Diabetic macula oedema	362.53, 362.07
Hypertension	401.xx-405.xx
Atrial fibrillation	427.31
Dyslipidemia	272.xx
Ischemic heart disease	410.xx-414.xx
Heart failure	428.xx
Chronic kidney disease	580.xx-589.xx, 403.xx-404.xx, 016.0x, 095.4x, 236.9x, 250.4x, 274.1x, 442.1x, 447.3x, 440.1x, 572.4x, 642.1x, 646.2x, 753.1x, 283.11, 403.01, 404.02, 446.21
Dialysis	585.xx (Catastrophic illness card)
Peripheral arterial disease	440.0x, 440.2x, 440.3x, 440.8x, 440.9x, 443.xx, 444.0x, 444.22, 444.8x, 447.8x, and 447.9x
Diabetic neuropathy	250.6x, 357.2x
Diabetic foot ulcer	250.7x, 785.4x
Vitreous haemorrhage	379.23, 362.81
Tractional retinal detachment	361.0x, 361.8x, 361.9x
Myocardial infarction	410.xx
Heart failure	428.xx
Ischemic stroke	433.xx, 434.xx, 435.xx, 436.xx, 437.xx
Haemorrhage stroke	430.xx, 431.xx, 432.xx

ICD-9 CM, International Classification of Diseases, Ninth Revision, Clinical Modification.
